# Modeling Cardiovascular Diseases with hiPSC-Derived Cardiomyocytes in 2D and 3D Cultures

**DOI:** 10.3390/ijms21093404

**Published:** 2020-05-11

**Authors:** Claudia Sacchetto, Libero Vitiello, Leon J. de Windt, Alessandra Rampazzo, Martina Calore

**Affiliations:** 1 Department of Molecular Genetics, University of Maastricht, Universiteitssingel 50, 6229ER Maastricht, The Netherlands; claudia.sacchetto.1@studenti.unipd.it (C.S.); L.deWindt@maastrichtuniversity.nl (L.J.d.W); 2 Department of Biology, University of Padova, via Ugo Bassi 58B, 35131 Padova, Italy; libero.vitiello@unipd.it; 3 Interuniversity Institute of Myology (IIM), Administrative headquarters University of Perugia, Piazza Lucio Severi 1, 06132 Perugia, Italy

**Keywords:** cardiac disease modeling, human induced pluripotent stem cells, 3D cardiac models, engineered heart tissue

## Abstract

In the last decade, the generation of cardiac disease models based on human-induced pluripotent stem cells (hiPSCs) has become of common use, providing new opportunities to overcome the lack of appropriate cardiac models. Although much progress has been made toward the generation of hiPSC-derived cardiomyocytes (hiPS-CMs), several lines of evidence indicate that two-dimensional (2D) cell culturing presents significant limitations, including hiPS-CMs immaturity and the absence of interaction between different cell types and the extracellular matrix. More recently, new advances in bioengineering and co-culture systems have allowed the generation of three-dimensional (3D) constructs based on hiPSC-derived cells. Within these systems, biochemical and physical stimuli influence the maturation of hiPS-CMs, which can show structural and functional properties more similar to those present in adult cardiomyocytes. In this review, we describe the latest advances in 2D- and 3D-hiPSC technology for cardiac disease mechanisms investigation, drug development, and therapeutic studies.

## 1. Introduction

Cardiovascular diseases (CVDs) are a major cause of morbidity and the first cause of death worldwide, and no decline in this trend is expected in the foreseeable future [1]. At present, the treatments of most cardiac disorders are still palliative and not really curative. Besides, in patients sharing similar symptoms, and hence classified as suffering from the same disease, the underlying molecular mechanisms can actually be different. For these reasons, the creation of new, human-relevant disease models is a task of paramount importance, as they would allow a better understanding of the onset, progression, and molecular mechanisms of the various diseases, paving the way for new therapeutic approaches.

Animal models have so far greatly contributed to the present knowledge on cardiac pathogenic mechanisms. Mice have played a key role in this field, thanks to the availability of ever more sophisticated methods of genetic manipulation, besides their ease of breeding compared to other mammalian models such as pigs. However, the murine heart exhibits important differences if compared with the human heart. Besides discrepancies in ion channel roles, calcium handling, and the development of cardiomyocytes, mice and humans present relevant difference in terms of cardiac electrophysiological properties, such as resting heart rate, repolarization phase of action potential, response to exercise, and duration of ventricular action potential [2,3]. These differences represent an obstacle to the creation of accurate models for human diseases and explain why many treatments proved inefficient when translated from murine preclinical studies to human clinical trials.

Primary cell lines derived from patients have been an important means to study cardiac diseases in vitro, and the above-mentioned issues related to animal models can be prevented by using human cardiomyocytes. However, heart primary cells show clear limitations, related to the invasive procedures required for their collection from patients and their limited survival and proliferation potential.

In the last decade, research focus has turned to strategies based on induced pluripotent stem cells (iPSCs) obtained from somatic cells. Human iPSCs (hiPSCs) were first described in 2007, when Takahashi and Yamanaka’s seminal work demonstrated the possibility to reprogram adult somatic cells to a pluripotent condition by overexpressing four transcription factors—OCT3/4, SOX2, KLF4, and c-MYC [4]. Due to their ability to potentially differentiate into cells of all three embryonic germ layers, hiPSCs showed soon their potential in the area of in vitro disease modeling, potentially offering numerous desirable features. First and foremost, thanks to their self-renewal capability, patient-derived hiPSCs could provide a potentially unlimited source of precursors carrying the genetic background of the patient, which could then be differentiated into a number of different tissues. Besides, as they can be obtained from somatic tissues, their use would allow us to bypass the ethical conundrums linked to the use of embryonic stem cells (ESCs).

In this review, we will focus on the application of hiPSCs in the context of two-dimensional (2D) and three-dimensional (3D) cardiac culture models for disease mechanisms investigation, drug development, and therapeutic studies.

## 2. Human iPSC-Derived Cardiomyocytes

**Figure 1:**
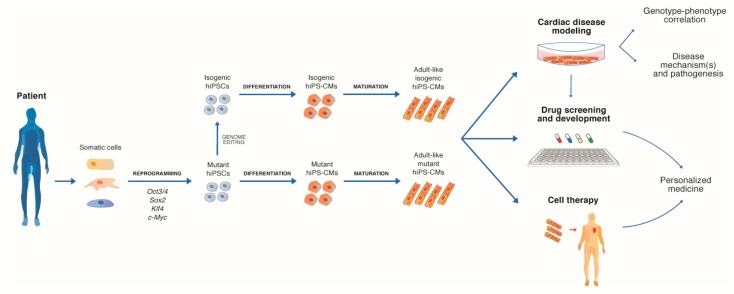
Schematic overview of patient-derived human-induced pluripotent stem cells–derived cardiomyocytes (hiPS-CMs) generation. The process consists of the following three main steps: reprogramming of somatic cells in human induced pluripotent stem cells (hiPSCs); hiPSCs differentiation in hiPSC-derived CMs; and maturation approaches to obtain adult-like hiPS-CMs. The resulting hiPS-CMs are ultimately used for research studies and clinical applications.

After the reprogramming of somatic cells and the validation of their pluripotent condition [5,6], hiPSCs can be differentiated into cells of mesodermal germ layer; this in turn can allow the generation of cardiac cell lineages, including cardiomyocytes (Figure 1). Floating embryoid bodies (EBs) or monolayer cultures on Matrigel are the two most commonly used methods to induce cardiac differentiation of hiPSCs [7]. In both cases, cells are placed in culture media containing defined sets of signaling molecules, specifically designed to induce cardiomyocytes (hiPS-CMs) generation.

### 2.1. 2D Cultures of hiPS-CMs for Disease Mechanisms Investigation

In the area of CVDs, hiPS-CMs have already shown a wide range of possible applications; important parameters like morphology, calcium handling, and contractility have been successfully analyzed in such systems. Studying hypertrophic cardiomyopathy (HCM), Lan et al. described the generation of patient-derived hiPS-CMs that displayed specific HCM-related alterations, such as abnormal sarcomere organization, cellular hypertrophy, and altered calcium handling [8]. In a different disease context, arrhythmogenic cardiomyopathy (ACM), transmission electron microscopy allowed to detect alterations of cell structure and morphology in hiPS-CMs obtained from patients carrying a plakophilin-2 (*PKP2*) gene mutation [9]. In these cells, the authors observed larger cardiomyocytes, altered Z-bands, and less organized desmosomes, hence recapitulating some typical ACM features [9]. Still in the context of ACM, abnormal plakoglobin nuclear localization was reported in patient-derived hiPS-CMs, together with decreased β-catenin activity in cardiogenic conditions [10]. In this work, the mutant hiPS-CMs showed increased lipogenesis and apoptosis as well as calcium-handling defects, once again recapitulating the disease phenotype and confirming that the hiPS-CM system could play a pivotal role in discovering ACM pathogenic mechanism(s) [10].

hiPS-CMs also offer an invaluable system for understanding the genetic basis of human CVDs and are being used as a model to test the pathological relevance of specific gene mutations. During the past few years, gene-editing approaches, such as TALENs and CRISPR/Cas9, have been used to correct suspected causative mutations in hiPSCs from patients [11,12,13]. This allowed not only the generation of proper experimental controls, i.e., isogenic hiPSC lines, but also the observation of a potential rescue of the defect caused by the mutation, thus suggesting its pathological relevance [14,15,16,17]. The opposite approach, i.e., the introduction of a mutation of interest in control hiPSC lines, has also been used [18,19,20,21,22,23,24]. Mosqueira and colleagues used CRISPR/Cas9 technology in three independent hiPSC wild-type lines to produce 11 variants of a suspected HCM-causing mutation, which they showed to promote severe and penetrant pathophysiology regardless of the genetic background [18]. Although Mendelian cardiac diseases are best suited for modeling through hiPS-CMs, Wei et al. recently reported interesting advances in recapitulating ischemic conditioning, suggesting the possibility to move toward the use of hiPS technology to also model non-Mendelian CVDs, such as myocardial infarction (MI) [25].

Human iPS-CMs have also allowed the investigation of the molecular pathways underlying CVDs [26,27,28]. The first example of such approach dates back to 2010, when Carvajal-Vergara and colleagues reported the generation of cardiomyocytes from patients suffering from “LEOPARD,” a complex syndrome that includes electrocardiographic abnormalities and hypertrophic cardiomyopathy [29]. In these cells, bFGF failed to activate MAPK, leading to an alteration of the RAS-MAPK signaling that in turn led to a HCM phenotype [29]. In another study, using hiPS-CMs from a patient with dilated cardiomyopathy (DCM) harboring a *TNNT2* mutation, the authors showed that an altered epigenetic regulation of key β-adrenergic signaling genes in mutant cardiomyocytes was the cause of contractile dysfunctions [30]. More recently, Lee et al. reported that the platelet-derived growth factor (PDGF) signaling pathway was abnormally activated in a patient-derived hiPS-CMs model of Lamin A/C-related DCM compared to isogenic controls, identifying a new potential target for therapeutic approaches [31]. Also, using patient-specific hiPS-CMs to model Duchenne muscular dystrophy (DMD) cardiomyopathy, single-cell RNA-seq analysis showed significant activation of fibrosis program in DMD hiPS-CMs compared to controls [32]. Moreover, combining DMD hiPS-CMs and human DMD left ventricle RNA-seq datasets, the authors found shared dysregulated pathways, suggesting the importance of this model for investigating cardiomyopathic mechanisms of DMD [32].

HiPS-CMs have also been used in electrophysiology studies based on patch clamp, calcium flux assays, and multielectrode arrays to describe aberrant features in genetic arrhythmic diseases, such as long QT syndrome (LQTS), catecholaminergic polymorphic ventricular tachycardia (CPVT), short QT syndrome (SQTS), sick sinus syndrome (SSS), as well as atrial fibrillation (AF) and Brugada syndrome (BrS) [33]. One of the first studies in which electrophysiological recording techniques have been used on hiPS-CMs was reported by Moretti at al., who found typical electrophysiological features of LQTS type 1 in patient-specific hiPS-CMs carrying a mutation in *KCNQ1* gene, which encodes the alpha subunits of the channels responsible for I_Ks_ generation [34]. More recently, electrophysiological studies were performed on hiPS-CMs derived from a SQTS patient carrying a mutation in the hERG potassium channel (*KCNH2*) responsible for the generation of the I_Kr_ current. This model recapitulated the disease phenotype, including shortened action potential duration and potassium current abnormalities [35]. The characterization of KCNH2 mutant hiPS-CMs was recently performed also in another study, confirming the ability of hiPSCs technology to provide important insights into arrhythmia mechanisms in SQTS [36].

### 2.2. 2D Cultures of hiPS-CMs as a Platform for Investigating CVDs Therapies

Another use of hiPS-CMs is the creation of disease models suitable for drug testing, as well as for drug discovery and development (Table 1) [37,38,39,40,41]. Particularly, hiPS-CM technology could be used to evaluate efficacy of drugs in a mutation-specific or even in a patient-specific context. One of the great opportunities provided by hiPS-CM is to establish large-scale, high-throughput screens for drug safety testing and discovery in large cohorts of patients. The validation and the use of these approaches for drug development is extremely attractive, as it could greatly reduce the costs resulting from animal disease models. One of the first studies demonstrating the reliability of hiPS-CM as a platform for drug evaluation was reported by Itzhaki et al. in the context of type-2 LQTS [42]. The authors assessed the effect of existing and novel pharmacological agents that could aggravate or ameliorate the disease phenotype, confirming the ability of the hiPS-CM model to recapitulate the expected drug response [42]. Studying the same cardiac disorder, Matsa et al. showed a proper response of LQT2 hiPS-CMs to clinically relevant pharmacology [43]. However, the two groups reported different responses to isoprenaline, likely because of different underlying mutations [42,43]. This discrepancy could support the potential use of hiPS-CMs from patients harboring different mutations in personalized medicine studies. More recently, Mehta et al. found that Lumacaftor, a drug acting as chaperone during protein folding, can correct the hERG trafficking defects in LQT2 hiPS-CMs, thus preventing arrhythmias in mutant cells [44]. These promising findings recently led to the realization of the first in-human study, highlighting the translational strength of this in vitro platform [45]. In the context of drug safety testing, Liang and colleagues for the first time recapitulated in hiPS-CMs drug-induced cardiotoxicity profiles for healthy subjects as well as for LQTS, HCM, and DCM patients, at the single cell level [46]. Not only healthy and diseased cells exhibited different susceptibilities to known cardiotoxic compounds, but hiPS-CMs could also accurately predict adverse drug responses [46].

Besides testing the effect of a specific compound, hiPS-CMs might be used also as a platform to develop new therapeutic strategies, including targeted genome editing [47], transgene overexpression [48], and RNA interference for either knockdown [49] or exon-skipping [50,51] in mutant genes.

The use of hiPS-CMs has been proposed in another, highly appealing research area, namely cell therapy for CVDs (Table 1). Considering the limited regenerative ability of the myocardium upon an adverse event such as a heart attack, hiPS-CMs may represent a potential solution to repopulate the damaged area. In this context, several studies have already been performed on small animals [60,61,62,63]. In 2015, hiPS-CMs transplanted into a mouse model after MI improved left ventricular (LV) function and attenuated cardiac remodeling [60]. Although engraftment was limited, transplanted hiPS-CMs provided pro-angiogenic and anti-apoptotic paracrine factors in an ischemic microenvironment [60]. The beneficial paracrine effects of hiPS-CMs on a murine injured myocardium have also been described by Tachibana et al., who observed no myocardial regeneration but cardio-protective paracrine effects on the damaged tissue [61]. Other studies reported grafting of transplanted hiPS-CMs. Transplantation of hiPS-CMs overexpressing the cell cycle regulator CCND2 in mice that underwent MI successfully improved the cardiac phenotype. Fifty percent of the myocardial scar was replaced over the following six months and LV function improved due to the electrical integration of the hiPS-CMs graft [62]. Large animals have also been used to test the potential of hiPSC technology in cell therapy for cardiac disorders [64,65,66,67]. Kawamura et al. generated hiPS-CMs sheets for transplantation over the MI areas in a porcine model of ischemic cardiomyopathy [64]. The authors observed attenuated LV remodeling and improved cardiac performance; however, very few hiPS-CMs survived long term [64]. In a more recent work, intra-myocardial injection of iPS-CMs in monkeys subjected to MI promoted cardiac contraction improvement for at least 12 weeks due to integrated graft survival [66]. As suggested by the above-mentioned works, cells survival in the host tissue is one of the main issues of hiPSC technology in cell therapy, and further studies will be needed to improve the hiPS-CMs engraftment efficiency and duration. In this context, the quality of the delivered cells can influence the efficacy of the treatment after hiPS-CMs transplantation. Current protocols used to prepare the cells for therapy consist of accelerated growth conditions, which lead to cell stress, including DNA damage. The quality of the cells can be selectively improved by activating transcription factor p53, which leads to apoptosis in DNA-damaged cells while not affecting DNA damage-free (DdF) cells [68]. This approach was used to prepare hiPS-CMs to treat heart failure caused by coronary artery ligation in mice. Cells were injected into the LV, and a significantly higher engraftment rate was found in murine hearts that received DdF hiPS-CMs when compared to controls, suggesting that the selected cells would better repopulate the ischemic myocardium of a failing heart [68].

### 2.3. Limits of hiPSC-Derived 2D Models and Current Strategies to Enhance hiPS-CMs Maturation

Despite the many progresses that have characterized the hiPSC field in recent years, such as the optimization of culture conditions allowing to obtain hiPS-derived cardiomyocytes with >90% purity [69], the use of hiPSCs in cardiac disease models still presents two major issues: the immaturity of hiPS-CMs and the inability of a 2D system to reproduce the complex 3D-structure of the heart tissue.

Van den Berg et al. showed that hiPS-CMs were comparable to first trimester gestational stage CMs in terms of structure, function and gene expression [70]. Indeed, hiPS-CMs are known to exhibit typical fetal-like features, such as reduced cellular size, immature myofibril alignment, lack of T-tubules, depolarized resting membrane potential, decreased ion channels expression and reduced upstroke velocity, fetal-like mitochondria, and metabolism [71]. Several approaches have been attempted to overcome the issue of hiPS-CMs immaturity; one of them is long-term culture. Kamakura et al. reported interesting ultrastructural sarcomeric changes in 180-day-old EB-contained cardiomyocytes compared to 14-day-old hiPS-CMs [72]. Specifically, in long-term culture, they observed myofibrils becoming more tightly packed and forming parallel arrays, together with the appearance of mature Z-, A-, H-, and I- bands; M-bands were also detected, but only in 360-day-old EBs [72]. The same structural improvements were observed by another group, who in addition performed electrophysiological assessments and found differences in AP amplitude, maximum diastolic potential, and upstroke velocity amongst 20/40-day-old and 80/120-day-old hiPS-CMs [73]. These findings suggest that hiPS-CMs are capable of maturing, albeit slowly, and better mimic the adult CMs phenotype. However, long-term culture is a cumbersome and expensive approach, which would be unsuitable for many experimental settings, such as high throughput screenings. Other strategies to promote hiPS-CMs maturation rely on the use of specific adult-like metabolic substrates, such as insulin and fatty acids [10,54,74], and of hormones and small molecules, including Tri-iodo-L-thyronine (T3) [75,76], GSKA [77], Wnt pathway modulators [78], or microRNAs [79,80,81]. Recently, inhibition of mTOR pathway has been found to enhance hiPS-CMs maturation [82]. Transient treatment during late differentiation with Torin1, a mTOR inhibitor, promoted a significant increased expression of mature cardiomyocyte markers in hiPS-CMs, as well as enhanced metabolic, contractile, and electrophysiological properties toward values observed in adult CMs [82]. Also, the modulation of the extracellular matrix (ECM) surrounding hiPS-CMs was reported to promote increased maturity in hiPS-CMs [83,84,85,86,87]. Matrix sandwiches combined with specific growth factors [83], as well as synthetic culture matrices engineered from combinatorial polymers [85], have been shown to enhance hiPS-CMs maturation and promote improvements in sarcomeric dimensions, mitochondrial function, electrophysiology, and contractility. However, there are still conflicting data about the optimal substrate properties. It was first reported that ESC- and hiPS-derived CMs maturation increased with substrate stiffness [86,88]. On the other hand, Herron et al. showed that culturing hiPS-CMs on Matrigel, and polydimethylsiloxane (PDMS), their softest experimental ECM condition, led to a higher expression and functional maturation levels compared to hiPS-CMs cultured on fibronectin and glass, their stiffest experimental ECM condition [87]. Another promising approach for hiPS-CMs maturation comes from the application of electric or mechanical stimulation, which can promote an appropriate physical modulation of the cellular microenvironment. Recently, physiological cyclic pulsatile hemodynamic forces were found to enhance hiPS-CMs maturation within a microfluidic system [89]. Particularly, the authors observed more elongated and rod-like-shaped hiPS-CMs with increased cell size, sarcomere length and alignment, contractility, and mitochondrial density, indicating an improved maturation of the cells when compared to static cultures [89]. Finally, the co-culture of hiPS-CMs with other cardiac cell types (e.g., fibroblasts or endothelial cells) has been also described as possible strategy to support cardiomyocytes maturation [90,91]. Yoshida et al. showed that co-culturing hiPS-CMs with mesenchymal stem cells (MSCs) can induce structural and functional maturation of hiPS-CMs in 2D culture [90]. MSCs were found to release soluble factors, including cytokines and exosomes, that promote the cardiomyocyte-specific markers expression impacting the maturation of hiPS-CMs [90].

The heart is characterized by a specific architecture in which cells are in contact with each other and the ECM. Despite the improved maturation protocols developed in the last few years, hiPS-CMs in 2D cultures are placed in a physiological and structural context that does not mimic the in vivo condition.

## 3. 3D Cultures of hiPS-CMs

**Figure 2:**
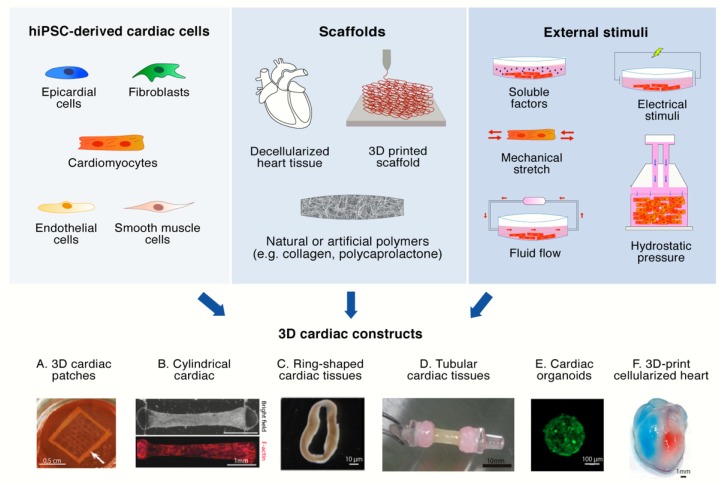
Generation of three-dimensional (3D) hiPSC-based cardiac constructs. Human iPSC-derived cardiac cells, scaffolds, and physical and environmental stimuli are the three main elements required for the generation of a 3D cardiac construct. Examples of 3D-hiPSC formats are reported from published studies. Figures reprinted from: (A) [93] Zhang et al., Tissue-engineered Cardiac Patch For Advanced Functional Maturation Of Human ESC-derived Cardiomyocytes, Biomaterials 34(23), 5813-20 (2013). With permission from Elsevier (Copyright 2013, Elsevier LTD.); (B) [111] Zhao et al., A Platform for Generation of Chamber-Specific Cardiac Tissues and Disease Modeling, Cell 176(4), 913-927 (2019). With permission from Elsevier (Copyright 2018, Elsevier Inc); (C) [109] Goldfracht et al., Generating ring-shaped engineered heart tissues from ventricular and atrial human pluripotent stem cell-derived cardiomyocytes, Nat Commun 11(1), 75 (2020). Licensed under the terms of the Creative Commons CC BY License (Copyright 2020, Springer Nature); (D) [112] Tsuruyama et al., Pulsatile tubular cardiac tissues fabricated by wrapping human iPS cells-derived cardiomyocyte sheets, Regen Ther 11, 297-305 (2019). With permission from Elsevier (Copyright 2019, The Japanese Society for Regenerative Medicine); (E) [113] Forsythe et al., Environmental Toxin Screening Using Human-Derived 3D Bioengineered Liver and Cardiac Organoids, Front Public Health 6, 13 (2018). Licensed under the terms of the Creative Commons Attribution License (CC BY) (Copyright 2018, Authors); (F) [114] Noor et al., 3D Printing of Personalized Thick and Perfusable Cardiac Patches and Hearts, Adv Sci (Weinh) 6(11), 1900344 (2019). Licensed under the terms of the Creative Common Attribution Licence (CC BY) (Copyright 2019, Authors).

In an effort to overcome the intrinsic limitations 2D cell cultures in terms of spatial architecture, recent years have seen a burgeoning development of three-dimensional culture models. Such phenomenon has also involved the field of heart diseases and many groups have developed 3D models of both healthy and pathological cardiac tissues. Once again, given the lack of *bona fide* adult precursors of cardiomyocytes, many of these systems have relied on hiPS-CMs. Importantly, several studies report that culturing hiPS-CMs within 3D constructs better recreates adult cardiomyocyte physiological, contractile, and electrical function, particularly when compared to 2D cultures [71,92,93,94,95,96,97,98,99] (Table 2). However, re-creating in vitro a reliable 3D tissue is undoubtedly more complex than using standard cell cultures, and several new aspects have to be considered simultaneously. Besides the source of cardiomyocytes, which has been discussed so far, at least three more elements must be considered for the creation of a 3D heart tissue model: the type of supporting scaffold, when present, the external stimuli that need to be applied and, last but not least, the role played by the non-contractile cell population(s) (Figure 2).

### 3.1. 3D Scaffolds

The peculiar organization of cardiac muscle fibers along specific directions, which define myocardial mechanical and physiological properties, is intrinsically linked to the ECM. Although present day in vitro 3D constructs cannot recreate anything similar to a whole heart, they do aim at reproducing as best as possible the in vivo cardiac tissue architecture; in this sense, scaffolds play a fundamental role in regulating cell adhesion, migration, proliferation and differentiation [115]. The molecular composition of the scaffold is a crucial feature in any 3D system, and the most obvious choice would then be to use the tissue’s own ECM. In 2013, Lu et al. generated a 3D heart model by combining functional hiPSC-derived cardiac progenitor cells and mouse decellularized heart matrix [116]. This 3D model exhibited the typical myocardium structure, the expected electrophysiological characteristics, and spontaneous contractions with a rate of 40–50 beats per minute, thus generating mechanical force [116]. Also, the engineered heart tissue showed normal response to various pharmaceutical agents that are known to affect cardiomyocyte physiology [116]. Importantly, the study reported the ability of ECM to promote hiPS-CMs differentiation, proliferation, and myofilament organization [116].

Another approach for fabricating in vitro 3D constructs relies on loading the desired cells into natural (e.g., gelatin, collagen, fibrin, alginate) [104,117] or artificial (e.g., polycaprolactone, poly D,L-lactic-co-glycolic acid) [118,119] polymers, which are sometimes combined together in hydrogel mixtures [120,121] and organized in oriented fibers [122,123] or in well-defined shapes [109,112]. Using hiPS-CMs from patients with Barth syndrome–related cardiomyopathy, Wang et al. succeeded in creating an engineered BTHS “heart-on-chip” tissue to simulate human myocardium, generating sheets of spontaneously beating cardiomyocytes [124]. Seeding hiPS-CMs onto thin elastomers micropatterned with fibronectin lines, hiPS-CMs self-organized into laminar myocardium that featured aligned sarcomeres [124]. Using a similar strategy, Zhao et al. generated a novel and open-access heart-on-a-chip system, in which hiPS-CM tissue contraction could be continuously monitored [125]. A “contactless” hydrodynamic approach, based on Faraday waves, has been used by Serpooshan et al. to promote rapid aggregation in a fibrin-based suspension of hiPS-CMs [102]. Such approach allowed the authors to reach cell packing densities that approximate native myocardium (10^8^–10^9^ cells/mL); further in vitro culture gave rise to formation of self-organized, closely packed, and symmetric 3D constructs, showing improved cell viability and maturation, intercellular connections, and contractile function when compared to constructs with random cell distribution [102]. The evidence that cell culture in 3D scaffolds promotes the maturation of hiPS-CMs has been recently confirmed by Silbernagel et al., who demonstrated for the first time that shaping iPSC-derived CMs in 3D micro-scaffolds can induce T-tubules formation, normally absent in 2D cultures, improving the structural and functional maturation of the cells [103].

In recent times, 3D bio-printing has also been used to recreate functional cardiac tissues based on stem-cell-derived CMs [105,114,126]. For example, Noor and colleagues succeeded in printing functional vascularized patches that matched the immunological, cellular, biochemical, and anatomical properties of the cell donor [114]. Specifically, after taking a biopsy of fatty tissue from patients, cells were extracted from part of the sample, while the remaining material was decellularized and processed to generate a fully personalized hydrogel, which served as a bioink for 3D printing [114]. Computerized tomography (CT) of a patient’s heart was used to identify the three-dimensional structure and orientation of the major blood vessel in the LV [114]. Afterward, anatomical data obtained from the CT images were used to design patch dimensions and blood vessel geometry, in order to generate a personalized scaffold [114]. hiPSC-derived cardiomyocytes and endothelial cells from patients were separately combined with the personalized hydrogel to form a bioink for the parenchymal cardiac tissue and blood vessels [114]. Moreover, since the patient-specific hydrogel could not sustain the weight of a whole printed organ, the authors used a printing strategy based on a support material composed of alginate microparticles in xanthan gum–supplemented growth medium, which maintained high cell viability and to print accurate, high resolution thick structures from the personalized hydrogel [114].

### 3.2. External Stimuli

Another limit of standard 2D hiPS-CM models is the difficulty to reproduce the in vivo–like condition in which stimuli coming from the external environment influence cardiac tissue development and maintenance. Indeed, a proper modeling of the myocardium should include not only electrical [125,127] but also mechanical [108] stimuli, such as those derived from fluid flow and hydrostatic pressure. Nunes and colleagues described an innovative platform for engineered heart construct in which a 3D cardiac tissue was generated by the self-assembly of hESC- and hiPSC-derived cardiomyocytes seeded into a template PDMS channel, around a surgical suture in type I collagen gels [100]. In this system, named “biowires,” cells were subjected to geometric electrical stimulation inducing highly organized cardiac structure and cell maturation. Indeed, their 3D constructs exhibited increased myofibril ultrastructural organization, enhanced calcium handling properties, and improved conduction velocity and electrophysiological properties compared to non-stimulated controls [100]. Thavandiran et al. generated aligned and functional 3D-hiPSC-derived cardiac microtissues, also defined as cardiac microwires (CMWs) [120]. In this work, pacing with point stimulation electrodes was found to promote cardiac maturation–associated gene expression and electrical signal propagation similar to in vivo conditions [120]. More recently, the Biowire II platform was used to generate 3D engineered cardiac tissues from hiPS-CMs and cardiac fibroblasts, employing long term electrical stimulation [128]. Thanks to the electrical conditioning, the engineered tissue expressed an adult-like human myocardium phenotype, including contractile properties and expected responses to therapeutic and to cardiotoxic agents affecting contractility [128].

Mechanical stimulation has also been proven to benefit 3D culturing of hiPS-CMs. Ruan et al. demonstrated that electric and mechanical stimulation could lead to the creation of advanced hiPS-derived cardiac microtissues, promoting the maturation of their structural, mechanical, and force generation properties after two weeks of electric pacing combined with static stress conditioning, achieved by maintaining constructs at a fixed static length [104]. Moreover, considering the positive effect on cardiac maturation of the increased mechanical loading during development, Leonard et al. tested the effect of moderate afterload on the maturation of hiPS-CMs in engineered heart muscles [129]. In this model, mechanical loading promoted increased hiPS-CM area and elongation, sarcomere length, and mRNA expression of maturation markers, as well as improved calcium handling and auxotonic contraction [129]. Similarly, Tsuruyama et al. found increased maturation in cardiac tubular 3D tissues in which hiPS-CMs were subjected to electrical and mechanical stimulation [112]. The authors observed increased expression of CM markers, including myosin light chain 2 (MYL2) and myosin light chain 7 (MYL7) when compared with hiPS-CMs cultured in static cultures [112].

### 3.3. Multi-Cellular Composition

Several lines of evidence showed the importance of multi-cellular interactions in promoting prolonged cell survival in 3D models [108,125]. As expected, the cellular composition of 3D cardiac tissues also plays a key role in better mimicking the physiology and the beating behaviour of native cardiac tissue [130,131,132]. Besides, mixed cell types composition in 3D cardiac constructs may improve drug testing, considering that different compounds can either act directly on CMs or indirectly, through the surrounding cells [133]. In recent years, various reports described methods to derive cardiac fibroblasts [134], smooth muscle [135,136], endothelial [137], and epicardial [138,139] cells from hiPSCs. However, primary cell lines might also be used in combination with hiPS-derived CMs. The possibility to generate different cardiac cell types offers the opportunity to model also those conditions in which the interaction between different cell types become decisive to exhibit the pathological phenotype [140], particularly characterized by the disruption of paracrine signals and cell-cell interactions. The combination of hiPS-CMs with fibroblasts has been reported to improve synchronised beating [141,142,143]. Jang and colleagues developed 3D cardiac macrotissues (CMTs) using a Layer-by-Layer (LbL) technique [144], based on deposition and centrifugation of hiPS-CMs and cardiac fibroblasts [145]. In this instance, the authors found that the incorporation of cardiac fibroblasts into the cardiomyocyte layer was a prerequisite for maturation and synchronized beating of CMTs, suggesting that the paracrine effects of fibroblasts could improve the functional properties of the CMT, compared to the mono-culture constructs [145]. Similarly, the introduction of endothelial cells in combination with hiPS-CMs has shown important advantages in the creation of advanced 3D cardiac constructs. For example, Giacomelli et al. generated human cardiac microtissues composed of hiPS-CMs and endothelial cells, finding increased expression of genes encoding cardiac ion channels and calcium handling proteins, which are considered evidences of advanced maturation when compared to hiPS-CM monoculture constructs [146].

Several groups described the generation of 3D cardiac constructs consisting on more than two cell types. Burridge et al. showed that in a 3D hydrogel tri-culture with hiPS-derived endothelial cells and human amniotic mesenchymal stem cells spontaneous synchronous contractility of hiPS-CMs was significantly increased when compared to hiPS-CM monoculture [147]. Amano and colleagues developed a vascularized 3D hiPS-CMs tissue by using a filtration layer-by-layer (LbL) technique for cells, showing that the introduction of cardiac microvascular endothelial cells together with human cardiac fibroblasts into the 3D hiPS-CM tissue modulated CMs organization and synchronous beating in 3D constructs; of notice, they also showed the formation of blood capillary-like networks [148]. Characterization of hiPS-CMs in tri-culture has been recently reported by Pitaktong et al., who observed improved microvasculature and increased contraction rate in 3D microtissue spheroids consisting of hiPS-CMs, adult cardiac fibroblasts, and hiPSC-derived early vascular cells, when compared with control 3D spheroids [149].

Importantly, hiPSC technology is also making progress toward the differentiation of cardiomyocyte subtypes: i.e., atrial, ventricular and nodal [150,151,152,153,154]. These CM subtypes are distinguished by electrophysiological properties and specific gene expression patterns [155,156]. Several groups have tried to modulate the latter to derive the different CMs subtypes, since early protocols for cardiac differentiation of hiPSCs resulted in heterogeneous populations of CMs, predominantly ventricular-like cells with a small percentage of atrial-like and nodal-like cells [155]. By combining stem-cell-derived CM differentiation protocols with electrical field conditioning, Zhao et al. were able to successfully model polygenic left ventricular hypertrophy, generating heteropolar cardiac tissues containing distinct atrial and ventricular ends [111]. These latter expressed chamber-specific genes and showed the expected drug responses [111]. Similarly, subtype-specific CMs differentiation protocols have been recently used to develop an engineered cardiac tissue that comprised chamber-specific human pluripotent stem-cell-derived cardiomyocytes (hPS-CMs) [109]. Ventricular and atrial hPS-CMs were embedded in a collagen-based hydrogel to generate ring-shaped 3D constructs showing proper atrial and ventricular phenotypes at gene and protein expression levels, as well as in terms of electrophysiological and contractile parameters [109]. However, none the above-described models recreated Purkinje fibers, the conduction system that electrically connect the atrial and ventricular chambers of the heart. The generation of an integrated cardiac tissue, including atrial and ventricular chambers, as well as vasculature network and electric conduction system, will likely further improve physiological-relevant drug response studies.

## 4. 3D Models for CVDs

### 4.1. 3D Cultures for Investigating CVDs Pathogenic Mechanisms

In some cardiac diseases the pathological phenotype is fully discernible only at a tissue/organ level [157], which makes 3D hiPS-CM models indispensable to avoid the use of animal models. For this reason, such systems are expected to soon become commonplace tools in the characterization of molecular mechanisms underlying cardiac diseases, as well as in preliminary testing and screening of drugs. In this regard, some brilliant works already succeeded in recapitulating cardiac organ functions and properties in a 3D model, both in healthy and pathological conditions (Table 3) [158,159,160,161]. Hinson and colleagues generated cardiac microtissues engineered from hiPS-CMs carrying different titin-truncating variants (TTNtvs), introduced by CRISPR/Cas9 technology [162]. The authors showed that some of the missense TTNtvs are pathogenic, leading to decrease contractile force in cardiac microtissues, whereas other variants promoted contractile function impairment only when genetic modifiers were also present [162]. Also, they observed that some of these variants produced a stable truncated protein unable to assemble with sarcomeric components, resulting in typical DCM patient features [162]. Stillitano and co-workers generated a 3D model starting from hiPS-CMs obtained from a patient harbouring a mutation in the phospholamban gene (*PLN*), shown to be causative of DCM [163]. A study performed by the same group in 2D hiPS-CMs from the same patient had already showed some features of DCM, such as calcium handling abnormalities and irregular electrical activity [14]; however, the 3D model demonstrated that the *PLN* mutation impaired cardiac contractility and that genetic correction restored contractile function [163]. In another study performed by Streckfuss-Bömeke et al., the authors obtained in parallel 2D hiPS-CM cultures and 3D cardiac constructs from a DCM patient, showing that the latter exhibited not only impaired force of contraction but also reduced passive stress of the tissue in response to gradual increase in strain, hence suggesting increased visco-elasticity of the mutated constructs [164]. The advantage of 3D cultures over monolayers in recapitulating disease features was also shown by Prondzynski and colleagues, who were able to detect some of the functional consequences of a α-actinin 2 (*ACTN2*) mutation only when hiPS-CMs derived from an HCM patient were cast in a 3D format [165]. The mutant hiPS-CMs embedded in the 3D construct showed increased contractility, relaxation deficit, higher myofilament Ca^2+^ sensitivity, and prolonged action potential duration when compared to controls, providing an in vitro model subsequently used to test personalized treatments for the patient [165].

Beside modeling inherited cardiomyopathies, 3D models could represent an attractive approach to study disease mechanisms underlying non-Mendelian cardiovascular disorders. This is made possible mostly by the introduction of advanced systems that manipulate specific parameters, e.g., oxygen content and medium composition, thus mimicking pathological conditions typical of disorders in which the genetic component is not the driving cause. Tiburcy et al. described an in vitro engineered human myocardium that showed structural and functional properties of postnatal tissue and could reproduce the typical hallmarks of heart failure upon chronic catecholamine overstimulation [99]. Recently, Sebastião et al. generated and characterized a new human in vitro 3D model of myocardial ischemia/reperfusion (I/R) injury using hiPS-CM aggregates and stirred tank bioreactors [166]. The extracellular microenvironment of I/R phases of acute myocardial infarction (MI) was recreated by finely controlling and monitoring critical process parameters, such as pO_2_ and pH [166]. The authors were able to mimic specific hallmarks of MI, including loss of CMs, cellular ultrastructure disruption, increased angiogenesis, and secretion of proinflammatory cytokines, suggesting that this model could serve as novel platform to investigate the disease mechanisms of MI [166]. Recently, Richards et al. recently generated 3D microtissues to mimic MI, establishing a model that combined an oxygen diffusion system with chronic adrenergic stimulation to create an apoptotic gradient in human cardiac organoids, which recapitulated the organotypic response of myocardium after infarction [167]. In this model, typical features of MI were recreated, including pathological calcium handling, metabolic shifts, and fibrosis [167]. Also, detrimental effects were observed in the 3D microtissues upon treatment with known cardiotoxic drugs, supporting the translational strength of this 3D model [167]. Moreover, in order to generate an in vitro model to study ventricular tachycardia, Lemme et al. investigated the effect of chronic tachypacing on hiPS-CMs embedded in a 3D engineered heart tissue [168]. The authors demonstrated a high vulnerability to tachycardia of tachypaced hiPS-CMs, terminated by ryanodine receptor stabilization or sodium or hERG potassium channel inhibition, hence indicating this new model as a potential tool to test anti-arrhythmic drugs to treat ventricular tachycardia [168].

### 4.2. 3D Models as a Platform for Developing Therapies for CVDs

In recent years, 3D cardiac models have been used in the context of drug screening and toxicity screening (Table 3). One of the many advantages of 3D models is the possibility to independently modulate molecular factors suspected of being involved in disease onset and progression by controlling, and measuring, the functional parameters of the tissue [177]. Huebsch et al. successfully developed miniaturized 3D cardiac tissues with the aim to perform physiologically relevant drug response analyses, once again showing that 3D constructs yielded more reliable results compared to 2D cultures [169]. Intriguingly, machine learning has been used by Lee et al. to analyze several different functional parameters obtained from force readouts of hiPSC-derived ventricular cardiac tissue strips embedded in a 3D collagen-based matrix and exposed to a library of compounds [170]. This way, the authors were able to generate a promising system for automated drug classification using a model capable of predicting the mechanistic action of an unknown drug [170]. Three-dimensional cardiac tissues derived from hiPS-CMs were also used as a platform for in vitro drug-induced cardiotoxicity assay [173]. Takeda et al. investigated the electrophysiological and contractile responses of the cardiac constructs challenged with cardiotoxic drugs known to promote different effects on CMs [173]. Dose-dependent cytotoxicity caused by doxorubicin was confirmed in the 3D cardiac tissues, as well as decreased hERG channel blocker-dependent beating rate, which instead was increased after isoproterenol treatment [173]. Importantly, the authors also reported that their model showed greater drug sensitivity than animal studies with the same compounds [173]. In yet another study, 3D human cardiac organoids were used to screen a panel of environmental toxins by assessing cell viability, ATP activity and organoid beating activity [113]. More recently, Mills et al. performed functional screening of more than 100 small molecules with presumed cardiac pro-regenerative potential using a high-throughput human cardiac organoid system [175]. This approach allowed to uncover detrimental side effects in many active molecules and led to the identification of two highly promising pro-proliferative molecules, as well as to the characterization of their mechanism(s) of action [175].

Altogether, these lines of evidence indicate that 3D models based on hiPSCs represent a viable alternative to at least part of the animal studies for the near future, thereby reducing research-related costs and potential failures in human clinical trials.

Last but not least, the idea of using 3D constructs based on hiPSCs for cell therapy in CVDs has been gaining traction for some years now. In the study by Shadrin and colleagues, smooth muscle cells and fibroblasts were combined with hiPS-CMs in a fibrin-matrigel scaffold, generating cardiopatches with clinically relevant size (4 × 4 cm) that exhibited electrical and mechanical functions similar to those observed in the adult human myocardium, including evidence for T-tubules and M-bands [171]. When implanted in nude mice via dorsal window chambers, cardiopatches underwent proper vascularization; when implanted onto rat epicardium, the same patches showed robust engraftment and maintained electrical function without increasing the incidence of arrhythmias [171]. Introducing the vascular component, Narita and co-workers also reported a hiPS-CM 3D tissue for therapeutic studies [172]. In their system they observed tubular structures consisting of endothelial cells organized around the hiPS-CMs, which provided a capillary network capable of supplying nutrients and oxygen throughout the tissue upon implantation on the LV of experimentally-infarcted rat hearts [172]. Besides leading to an overall higher survival rate, the presence of the engrafted tissues led to a significant increase in wall thickness and to a wide distribution in the implanted area of functional blood vessels, comprising both host and implanted endothelial cells [172]. Driven by the same aim, Mattapally et al. produced hiPS-CM spheroids, embedded in a fibrin patch, for transplantation in mice after experimentally-induced MI [174]. Patch-receiving mice showed a higher engraftment rate and improved cardiac function compared to non-treated mice [174]. More recently, Wanjare et al. generated spatially patterned myocardial tissues combining hiPSC-derived cardiomyocytes and endothelial cells seeded in microfibrous polycaprolactone scaffolds [176]. When transplanted in a rat myocardial injury model, the engineered tissues promoted pro-survival and pro-angiogenic effects [176]. Interestingly, the authors found that randomly oriented scaffolds promoted microvessel formation and higher arteriole density compared to engineered tissues with aligned scaffolds, underlying how scaffold topography can play a key role in promoting differential effects on cellular survival and revascularization [176].

## 5. Conclusions and Perspectives

In the last decade, the emergence and growth of hiPSC technology has led to countless progress in the medical field and the generation of diseased hiPS-CMs has already provided many advances in the understanding of the genetic and molecular pathophysiology of cardiac disorders.

However, differentiation of hiPS-CMs in 2D cultures yields inadequate cell maturation, representing a major obstacle to disease modeling and clinical translations of cell therapies. More efforts should be dedicated to fine-tuning hiPS-CMs maturation, e.g., by applying combinatorial approaches and coordinating the timing and the intensity of a specific intervention to recreate the natural cardiac developmental program. On the other hand, development of 3D cardiac models is already showing improvements in hiPS-CMs maturation due to the multi-cellularity of these systems and to their ability to reproduce the physical and environmental cues necessary for the physiological maturation process from postnatal stages into adulthood. However, a fully mature cardiac tissue that recapitulates all the properties of an adult heart has not yet been generated, and this gap might be filled only by uncovering the molecular mechanisms that govern postnatal maturation of the human heart, including those deriving from postnatal inter-organ communication. Mature hiPS-CMs are essential to model typical adult-onset disorders or conditions in which the pathogenic mechanisms involve mechanisms or cellular components that are observed only in mature cells. However, while the presence of hiPS-CMs with an embryonic or fetal phenotype is considered to be a limitation in most cases, such cells might be required for cell therapy applications, in which a partially differentiated phenotype is conducive to better engraftment and proliferation of hiPS-CMs delivered in infracted myocardium [178]. So far, several approaches have been used in the generation of cardiac 3D models—namely, bioreactors, biomimetic scaffolds, 3D bioprinting technologies, and organ-on-a-chip micro-physiological systems. Each technique allowed the implementation of specific features, such as electrical stimulation, mechanical loading, fine-tuning of medium composition, and oxygen content. However, the increased complexity of these 3D systems could make them less widely accessible. In terms of scalability, different applications require constructs of different size; larger formats are mostly necessary for heart regeneration, whereas miniaturized 3D systems are mainly needed for basic research or large-scale drug screening. Different sizes then imply different levels of complexity; for example, large formats also require systems that provide sufficient nutrient and oxygen supply to avoid the formation of necrotic areas. The number of cells required for a 3D construct is another important factor to consider, as an average of 0.5–2 million cardiomyocytes per tissue are usually necessary for 3D systems. Such requirement explains the work being carried out toward cell number optimization and the creation of smaller functional tissues comprised of fewer cells, thus reducing time and costs of production. Therefore, depending on the required application, the challenge will be to generate advanced 3D hiPS-CM platforms capable of providing relevant microenvironment cues combined with a relative ease of use.

Although technology of 3D models is still in its infancy and further advances in hiPSC culture systems will be necessary (e.g., in biomaterial, microfluidics and mechanical engineering, as well as in microfabrication methods), the new platforms based on 3D models are already leading to better high-throughput screening of new molecules for therapeutics. In turn, these approaches are generating larger multidimensional datasets, which will require the development of new methods to fully characterize drug responses. Importantly, 3D humanized in vitro platforms could acquire a pivotal role in the area of personalized medicine, potentially providing patient-specific in vitro drug screening and therapeutic approaches, once technological advances will have reduced the time and cost factors to manageable scales. Finally, despite the achievement of crucial goals in hiPS-CMs differentiation and maturation, as well as in 3D construct generation, it should be noticed that the growing body of work we are now witnessing at times shows conflicting data, likely due to the lack of hiPS-CMs quality control and standardized experimental conditions among different studies. In this regard, a fitting example is represented by electrophysiology studies; in this context, there are examples of hiPS-CMs showing a lack of ion currents normally observed in adult cells [87,179], but also of hiPS-CMs expressing ion channels that are absent in adult cardiomyocytes [110,180]. This consideration underlines the importance of carefully evaluating the expression of cardiomyocyte-specific ion channels in hiPS-CMs before considering a particular model for electrophysiology and/or therapeutic studies. Therefore, a major challenge now is to develop standardized protocols for reproducible production of high-quality hiPS-CMs, to be used for studying CVD and for possible clinical applications. Considering the advances obtained within the last decade, these goals might not be too far in the future.

## Figures and Tables

**Table 1:** List of 2D cardiac disease models for pharmacological studies described in this review.

Ref.	Objective	Pharmacological Approach and Outcome of the Study
[42]	LQTS type 2 treatment	- E-4031 and cisapride → pro-arrhythmic effect - Nifedipine → shortened APD_90_, elimination of EAD events, abolished arrhythmic events, long-term treatment associated with toxicity - Pinacidil → APD_90_ shortening, abolished EADs, potentially pro-arrhythmic - Ranolazine → anti-arrhythmic effect
[43]	LQTS type 2 treatment	- E-4031 → APD/FPD prolongation, EADs (only in mutant CMs) - Nicorandil and PD-118057 → shortened APD, abolished EADs
[52]	Drug safety study	Confirmed effect of 25 known cardioactive compounds
[37]	CPVT	- Dantrolene → rescue of the arrhythmogenic defect (normal Ca^2+^ spark properties)
[46]	Cardiotoxicity study for LQTS, HCM, and DCM	- Cisapride → pro-arrhythmic effects (particularly in LQTS and HCM) - Nicorandil → normalized APD and abolished EADs (LQTS). Dose-dependent pro-arrhythmic effect
[53]	Drug safety study (10 compounds)	- E-4031 and Cisapride → prolonged FPD and pro-arrhythmic effect - Nifedipine and Verapamil → dose-dependent FPD shortening, increased beat rate - Terfenadine → dose-dependent effect on FPD, no pro-arrhythmic effect - Quinidine, Mexiletine → reduced spike amplitude - Flecainide → reduced spike amplitude and pro-arrhythmic potential - GSK A → dose-dependent FPD prolongation - GSK B → increased heart rate
[38]	JLNS treatment	- Noradrenaline → Increased APD_90_ and PlaA - Cisapride → genotype-dependent pro-arrhythmic effect - NS1643 → reduced FPD and protection from cisapride-induced arrhythmias
[22]	LQTS treatment	- Nifedipine → APD_90_ shortening - Pinacidil → APD_90_ shortening
[54]	Diabetic cardiomyopathy treatment	Screening of 480 compound for CM phenotype preservation during diabetic stress: - Thapsigargin and Fluspirilene → Identified as most-effective compounds
[55]	Drug safety study (24 compounds)	16 of 18 compound with known clinical cardiac risk showed drug-induced effect in hiPS-CMs upon structural and functional evaluation
[56]	Drug safety study	Bay K8644, Mibefradil, NS1643, Levcromakalim, Ouabain → Repolarization effects Isoproterenol, ZD7288, BaCl2 → Chronotropic effects Quinidine, Cisapride, Thioridazine, Astemizole, Bepridil, Pimozide → Arrhythmogenic effect Amiodarone, Tolterodine, Vanoxerine, Alfuzosin, Ranolazine → FPD prolongation (clinical QT prolongation)
[44]	LQTS type 2 treatment	-Lumacaftor → shortened FPD, genotype-dependent hERG membrane localization, increased I_Kr_ current density, reduced Ca^2+^-handling abnormalities
[57]	Drug safety study	Screening of 28 known pro-arrhythmic drugs: all the analysed compounds confirmed pro-arrhythmic effect
[58]	Preventing oxidative injury post MI	Screening of 48,649 protective molecules preventing peroxide-induced cell death: - Cardioprotectant 312 (CP-312) → increased antioxidant response
[39]	DCMA syndrome treatment	SS-31 → reduced mitochondrial fragmentation
[59]	Cardiomyocyte protection in MI	Screening of 1800 active compounds: - F1386-0303 → suppressed hiPS-CMs death

APD, action potential duration; APD_90_, action potential duration at 90% repolarization; CM, cardiomyocyte; CPVT, catecholaminergic polymorphic ventricular tachycardia; DCM, dilated cardiomyopathy; DCMA, dilated cardiomyopathy with ataxia; EAD, early after depolarization; FPD, field potential duration; HCM, hypertrophic cardiomyopathy; hiPSC; human induced pluripotent stem cell; hiPS-CM, human induced pluripotent stem cell-derived cardiomyocyte; JLNS, Jervell and Lange-Nielsen syndrome; LQTS, long QT syndrome; LVEF, left ventricular ejection fraction; MI; myocardial infarction; PlaA, plateau amplitude.

**Table 2:** Improved maturation of hiPS-derived cardiomyocytes within 3D constructs.

Feature	Phenotype of hiPS-CMs in 3D System	References
Proliferation	Decreased proliferation capacity	[100,101]
Morphology	Switch from a round/polygonal to a more rod-shaped morphology	[95,100,102,103]
Cell size	Increased cell area and volume	[92,100,103,104,105,106]
Number of nuclei	Multinucleation	[105,106]
Cell aggregation	hiPS-CMs are robustly interconnected by electrical and mechanical junctions	[93,100,102,104,106,107]
Contractile apparatus	Increased sarcomere organization, length, and alignment Early-stage t-tubules formation	[93,95,100,102,103,104,106,108] [103]
Mitochondria	Increased number of mitochondria close to the contractile apparatus	[100,103,108]
Gene expression	Decreased expression of: - fetal cardiac genes Increased expression of: - ion channel genes of mature CMs - cardiac contractile function and sarcomere-related genes (*MYL2*, *MYOZ2*, *TCAP*, *MYL3*, *MYOM2*, *MYLK3*, *MYH7B*, *MYH6*, *TNNI3*, *TNNT2*, *ACTC1*)	[92,93,100] [93,100] [92,93,101,102,105,107,108]
Cardiac contraction	Increased contraction force	[93,102,104,106,107]
Cardiac conduction	Increased conduction velocity	[93,100,102,106,107,109]
Calcium handling	Increased expression of calcium-handling genes (*SERCA*, *RYR2*, *CX43*, *ASPH*, *CaV1.2*, *NCX1*, *HCN4*, *CASQ*2, *TRDN*) Improved calcium transient amplitude	[93,104,105,107]; [94,103,105,106,110]
Electrophysiological properties	Resting membrane potential (V_rest_) closer to adult CMs Increased APD_50_ and APD_90_ Increased maximum upstroke velocity (higher INa density)	[100] [92,94] [92,95]
Response to β-adrenergic stimulation	Increased adrenergic response to catecholamine stimulation	[93,107,109,110]
Metabolism	Higher oxygen consumption rate (OCR) Increased expression of fatty acid oxidation-related genes Decreased expression of glycolytic genes	[92]

APD_50_, action potential duration at 50% repolarization; APD_90_, action potential duration at 90% repolarization; CM, cardiomyocyte; hiPSC, human induced pluripotent stem cell; hiPS-CM, human induced pluripotent stem cell-derived cardiomyocyte.

**Table 3:** List of 3D hiPSC-based cardiac constructs reported in this review.

Ref.	Scaffold	Cell Types	Physical Stimulation	Disease Modeling	Therapeutic Studies
[108]	Type I Collagen-based 3D scaffold	- hESC-/hiPS-CMs (2 × 10^6^) - HUVEC (1 × 10^6^) - human MSCs/MEFs (1 × 10^6^)	Uniaxal cyclic stress conditioning (mechanical)	N/A	Implantation in athymic rats
[93]	3D cardiac patches (PDMS + fibrinogen, Matrigel, thrombin)	hESC-CMs (1 × 10^6^)	Patches cultured on a rocking platform	N/A	N/A
[116]	Decellularized mouse heart	hiPSC-derived MCPs (CMs, SMCs, ECs) (Tot. 1 × 10^7^)	N/A	N/A	Drug responsiveness
[120]	Collagen master mix (Collagen I and Matrigel)	- hESC-CMs - hiPS-derived FBs (Tot. 0.5 × 10^6^)	Uniaxial mechanical stress and electrical point stimulation	Tachycardic model of arrhythmias	Drug responsiveness
[100]	Type I collagen gel	hESC-/hiPS-derived MCPs (CMs, ECs, FBs, SMCs)	Electrical stimulation	N/A	N/A
[124]	Elastomers micropatterned with fibronectin lines	hiPS-CMs (1 × 10^5^ cells/cm^2^)	N/A	Barth syndrome cardiomyopathy caused by *TAZ* mutations	N/A
[147]	3D Hydrogel platform (Matrigel-based)	- hESC-CMs (2.5 × 10^5^) - hiPSC-derived ECs (5 × 10^4^) - hAMSCs (5 × 10^4^)	N/A	N/A	N/A
[162]	PDMS substrate + collagen I and human fibrinogen	- hiPS-CMs - Human MSCs (Tot. 1.1 × 10^6^)	Electrical field stimulation	DCM caused by *TTN* mutations	N/A
[104]	Collagen (I)-based 3D scaffold	hiPS-CMs (2 × 10^6^)	Electric pacing and static stress conditioning	N/A	N/A
[130]	Matrigel + collagen type I matrix	- hiPS-CMs - hiPSC-derived ECs - hiPSC-derived MCs (Tot. 3 × 10^6^)	N/A	N/A	Implantation in a rat MI model
[148]	Fabricated fibronectin and gelatin nanofilms (LbL assembly)	- hiPS-CMs - Human cardiac FBs (Tot. 1–50 × 10^5^)	N/A	N/A	Responsiveness to cardiotoxic drugs
[158]	Micropatterned wells (PDMS substrate + collagen type I and fibrinogen)	- hiPS-CMs - human mesenchymal stem cells (Tot. 1.1 × 10^6^)	N/A	PRKAG2 cardiomyopathy	N/A
[160]	PDMS template + Matrigel/collagen I matrix	- hiPS-CMs - stromal cells (Tot. 1 × 10^6^)	N/A	HCM caused by *BRAF* mutation	N/A
[163]	PDMS template + Matrigel/collagen I matrix	hiPS-CMs (1 × 10^6^)	N/A	DCM caused by *PLN* mutation	N/A
[169]	PDMS stencils containing rectangular through-holes (hydrogel-free)	- hiPS-CMs - hiPSC-derived FBs (Tot. 2 × 10^7^)	N/A	N/A	Clinically relevant responsiveness to isoproterenol treatment
[95]	Agarose casting molds + fibrin matrix (Matrigel, fibrinogen, thrombin)	hiPS-CMs (1 × 10^6^)	N/A	N/A	N/A
[99]	Circular casting molds + Matrigel/collagen matrix	- hESC-/hiPS-CMs - human FBs (Tot. 1 × 10^4^–15 × 10^6^)	Dynamic stretch conditioning	Model of heart failure	Implantation in athymic rats
[102]	Fibrin hydrogel suspension (fibrinogen, thrombin)	hiPS-CMs (1.7 × 10^6^)	Hydrodynamic drag force fields (Faraday waves)	N/A	N/A
[105]	3D-MPE printed scaffold (methacrylated gelatin-based)	- hiPS-CMs (25 × 10^3^) - hiPSC-derived ECs (12.5 × 10^3^) - hiPSC-derived SMCs (12.5 × 10^3^)	N/A	N/A	Implantation in a murine model of MI
[131]	PDMS template + Matrigel/collagen I matrix	- hiPS-CMs - hiPSC-derived ECs - hiPSC-derived MCs (Tot. 6 × 10^6^)	N/A	N/A	Implantation in rat model of MI
[146]	Scaffold-free (spheroids)	- hESC-/hiPS-CMs - hESC-/hiPSC-derived ECs (Tot. 5 × 10^3^)	N/A	N/A	Drug responsiveness
[164]	Circular casting molds + Matrigel/collagen matrix	hiPS-CMs (1 × 10^6^)	Mechanical stress conditioning	DCM caused by *RBM20* mutation	N/A
[170]	PDMS elastomer molds + Matrigel/collagen I matrix	- hESC-CMs (1 × 10^5^) - human FBs (1 × 10^5^)	N/A	N/A	Responsiveness to cardioactive drugs
[171]	PDMS square molds + Hydrogel matrix (fibrinogen, Matrigel, thrombin)	hiPS-CMs (0.5–1 × 10^6^)	Patches cultured on a rocking platform	N/A	Implantation in nude mice (skin) and nude rats (epicardium)
[172]	Fabricated fibronectin and gelatin nanofilms (LbL assembly)	- hiPS-CMs - human cardiac FBs - HCMVECs (Tot. 1.1–3.7 × 10^6^)	N/A	N/A	Implantation in rat infarcted hearts
[97]	PDMS elastomeric pillars + fibrin hydrogel (fibrinogen, thrombin)	- hiPS-CMs - human dermal FBs (Tot. 2 × 10^6^)	Mechanical loading and electrical conditioning	N/A	Responsiveness to isoproterenol treatment
[123]	PDMS elastomer molds + collagen I matrix	- hiPS-CMs (1 × 10^6^) - human MSCs (0.2 × 10^6^)	Static stress conditioning	Systolic cardiomyopathy (*MYH7* mutation)	N/A
[129]	Rectangular agarose/PDMS casting molds + fibrinogen/thrombin matrix	- hiPS-CMs (5 × 10^5^) - human MSCs (5 × 10^4^)	Mechanical loading (afterload)	N/A	N/A
[161]	Cell-encapsulation gel-free filamentous matrix (OrmoClear^®^polymer)	- hiPS-CMs - hiPSC-derived FBs - hiPSC-derived stromal cells (Tot. 3 × 10^6^)	Mechanical conditioning	Contractile dysfunctions caused by *MYBPC3* deficit	N/A
[173]	Fibronectin/gelatin nanofilms (LbL assembly)	- hiPS-CMs - hiPSC-derived FBs - hiPSC-derived SMCs (Tot. 5 × 10^5^)	N/A	N/A	Drug-induced cardiotoxicity assay
[113]	Scaffold-free (organoids)	- hiPS-CMs - human cardiac FBs (Tot. 1 × 10^3^)	N/A	N/A	Environmental toxin screening
[174]	Fibrin matrix (patch) containing spheroids (spheroid fusions)	hiPS-CMs (2.5 × 10^3^–3 × 10^5^)	N/A	N/A	Implantation in a murine model of MI
[112]	Cell sheets wrapped around a hollow octagonal tubular column (fibrin and collagen gels to seal the extremities)	- hiPS-CMs (6 × 10^6^ cells/sheet) - human dermal FBs (6 × 10^6^ cells/sheet)	Electrical stimulation and mechanical stretch (provided by a circulation system)	N/A	N/A
[114]	Personalized hydrogel from decellularized human ECM	- hiPS-CMs (1 × 10^8^) - hiPSC-derived ECs (1.5 × 10^7^) - human neonatal dermal FBs (3 × 10^6^)	N/A	N/A	N/A
[128]	Parallel POMaC wires + hydrogel matrix (collagen, Matrigel, fibrin)	- hiPS-CMs (1 × 10^5^) - human cardiac FBs (1 × 10^4^)	Long-term electrical field stimulation	N/A	Canonical responses to cardiotherapeutic and cardiotoxic agents
[111]	Flexible POMaC wires + hydrogel matrix (collagen, Matrigel)	- hESC-/hiPS-CMs (7.47 × 10^4^) - human cardiac FBs (3.5 × 10^4^)	Electrical field stimulation	Left ventricular hypertrophy	Chamber-specific responsiveness to serotonin and ranolazine
[159]	Micromolded gelatin muscular thin film substrate + fibronectin and gelatin matrix	hiPS-CMs (1 × 10^6^)	N/A	CPVT caused by *RYR2* mutation	N/A
[165]	Flexible PDMS posts + fibrin matrix (Matrigel, fibrinogen, thrombin)	hiPS-CMs (8 × 10^5^)	N/A	HCM caused by *ACTN2* mutation	Ameliorated phenotype upon diltiazem treatment
[168]	Agarose/PDMS-casting molds + fibrin matrix (Matrigel, fibrinogen, thrombin)	hiPS-CMs (1 × 10^6^)	Chronic optical tachypacing	Ventricular tachycardia	Responsiveness to antiarrhythmic compounds
[175]	PDMS molds + Matrigel/Collagen I mixture (to form cardiac organoids)	- hESC-CMs - hESC-derived stromal cells (Tot. 5 × 10^4^)	N/A	N/A	Functional screening of 105 compounds with pro-regenerative potential
[176]	Circular microfibrous polycaprolactone sheets + Geltrex	- hiPS-CMs (1 × 10^6^) - hiPSC-derived ECs (4 × 10^4^)	N/A	N/A	Subcutaneous transplantation in SCID mice and epicardial transplantation onto rat MI model
[109]	Ring-shaped casting molds + Collagen-based hydrogel matrix	- hESC-CMs (atrial and ventricular differentiation) (Tot. 2 × 10^6^)	Passive stretch conditioning	Atrial arrhythmia model (AF-like)	Arrhythmic phenotype rescued after electrical cardioversion or after treatment with anti-arrhythmic agents
[125]	Polystrene chip with two parallel POMaC wires + collagen hydrogel (with Matrigel) or collagen/fibrin hydrogel (with fibrinogen)	- hESC-/hiPS-CMs (atrial and ventricular) - mesenchymal stem cells - human cardiac FBs (Tot. 25–150 × 10^6^)	Electrical conditioning	N/A	N/A
[103]	3D-printed cuboids and hexagons micro-scaffolds (PETA monomer)	-iPSC-derived CMs (murine) (2 × 10^4^)	N/A	N/A	N/A
[145]	PDMS support + collagen I mixture (LbL assembly)	- hESC-/hiPS-CMs (1 × 10^6^) - human cardiac FBs (2 × 10^5^)	N/A	N/A	N/A
[149]	Scaffold-free (3D-bioprinted spheroids)	- hiPS-CMs - human cardiac FBs - HUVEC or hiPSC-derived vascular cells (Tot. 3.3 × 10^4^)	N/A	N/A	N/A
[166]	Scaffold-free aggregates (3D self-organization)	hiPS-CMs (1.5 × 10^3^)	3D aggregates cultured with controlled pO_2_ and pH in stirred-tank bioreactors	Myocardial ischemia-reperfusion injury model	N/A
[167]	Scaffold-free self-assembled organoids	- hiPS-CMs - human cardiac FBs - HUVEC - human adipose-derived stem cells (Tot. 1.5 × 10^5^)	Oxygen-diffusion gradient	Model of MI	Fibrotic phenotype ameliorated upon treatment with HF drug candidate and canonical cardiotoxic response to doxorubicin

+, combined with; 3D, three-dimensional; ACTN2, alpha actinin 2; AF, atrial fibrillation; CM, cardiomyocyte; CMPC, cardiomyocyte progenitor cell; DCM, dilated cardiomyopathy; EC, endothelial cell; ESC, embryonic stem cell; FB, fibroblast; hAMSC, human amniotic mesenchymal stem cell; HCMVEC, human cardiac microvascular endothelial cells; hESC-CM, human ESC-derived cardiomyocyte; HCM, hypertrophic cardiomyopathy; HF, heart failure; hiPSC, human induced pluripotent stem cell; hiPS-CM, human induced pluripotent stem cell-derived cardiomyocytes; HUVEC, human umbilical vein endothelial cell; LbL, Layer-by-Layer; MC, mural cell; MCP, multipotential cardiovascular progenitor; MEF, mouse embryonic fibroblast; MI, myocardial infraction; MPE, multiphoton excited; MSC, marrow stromal cells; MYBPC3, myosin binding protein C3; MYH7, myosin heavy chain 7; PDMS, polydimethylsiloxane; PETA, pentaerythritol triacrylate; pO_2_, partial pressure of oxygen; PLN, phospholamban; POMaC, poly (octa-methylene maleate (anhydride) citrate); PRKAG2, protein kinase AMP-activated non-catalytic subunit Gamma 2; RBM20, RNA-binding motif protein 20; RYR2, ryanodine receptor 2; SCID, severe combined immunodeficiency; SMC, smooth muscle cell; TAZ, tafazzin; Tot., total number of cells; TTN, titin.
